# Numerical Transcoding Proficiency in 10-Year-Old Schoolchildren is Associated with Gray Matter Inter-Individual Differences: A Voxel-Based Morphometry Study

**DOI:** 10.3389/fpsyg.2013.00197

**Published:** 2013-04-25

**Authors:** Amélie Lubin, Sandrine Rossi, Grégory Simon, Céline Lanoë, Gaëlle Leroux, Nicolas Poirel, Arlette Pineau, Olivier Houdé

**Affiliations:** ^1^Laboratory for the Psychology of Child Development and Education, Sorbonne, CNRS, Unit 3521Paris, France; ^2^Sorbonne-Paris-Cité Alliance for Higher Education and Research, Paris Descartes UniversityParis, France; ^3^Normandy Alliance for Higher Education and Research, Caen UniversityCaen, France; ^4^CNRS, UMR 5296 GINBordeaux, France; ^5^CEA, UMR 5296 GINBordeaux, France; ^6^University of Bordeaux, UMR 5296 GINBordeaux, France; ^7^Institut Universitaire de FranceParis, France

**Keywords:** mathematics learning, number processing, educational neuroscience, schoolchildren, gray matter, voxel-based morphometry, neuroeducation

## Abstract

Are individual differences in numerical performance sustained by variations in gray matter volume in schoolchildren? To our knowledge, this challenging question for neuroeducation has not yet been investigated in typical development. We used the Voxel-Based Morphometry method to search for possible structural brain differences between two groups of 10-year-old schoolchildren (*N* = 22) whose performance differed only in numerical transcoding between analog and symbolic systems. The results indicated that children with low numerical proficiency have less gray matter volume in the parietal (particularly in the left intraparietal sulcus and the bilateral angular gyri) and occipito-temporal areas. All the identified regions have previously been shown to be functionally involved in transcoding between analog and symbolic numerical systems. Our data contribute to a better understanding of the intertwined relationships between mathematics learning and brain structure in healthy schoolchildren.

## Introduction

How are individual differences in numerical cognition associated with structural brain variations in schoolchildren? Not only developmental maturation but also experience and learning may modify brain structure. On one hand, brain maturation is characterized by loss of gray matter (GM) with age that varies according to brain region (Gogtay et al., 2004). On the other hand, experience-dependent structural plasticity was demonstrated by an increase of GM in cerebral areas associated with training function in expert groups (e.g., mathematicians, Aydin et al., 2007) and was demonstrated during intensive trainings (e.g., motor learning, Draganski et al., 2004; or cognitive training, Draganski et al., 2006). The aim of the present study is to provide insights into the relationships between individual differences in mathematics and brain structure (GM volume variations) in 10-year-old typically achieving schoolchildren. As suggested by Carew and Magsamen (2010), linking brain research to education is extremely important for a better understanding of how children learn. It seems central that studies concerning brain plasticity, such as our study, bring information to the educational community in order to contribute to a better quality of education and an adapted pedagogy. However, direct applications within the classroom were still difficult to consider (Hook and Farah, 2012).

Numerical cognition is fundamental for everyday life activities, arises early in development (Feigenson et al., 2004; Cordes and Brannon, 2008) and improves significantly with the emergence of symbolization thanks to language acquisition and academic learning (Gelman and Butterworth, 2005). During schooling, children learn a wide range of mathematics skills. For instance, the first mathematics learning involves associating a quantity (analog system) with its symbol (oral or written numerical word, hereafter called the symbolic system). This transcoding between analog and symbolic systems is essential for developing sophisticated numerical tools (Holloway and Ansari, 2009; Mundy and Gilmore, 2009).

Numerical cognition has been extensively investigated in functional Magnetic Resonance Imaging (MRI) in children (e.g., Ansari et al., 2005; Ansari and Dhital, 2006; Cantlon et al., 2006; Kaufmann et al., 2006). Recent functional MRI meta-analyses collected these studies and indicated that numerical cognition mainly involved a frontoparietal network constituting the right inferior and the left superior frontal gyri and the upper part of the left middle occipital gyrus near the junction with the parietal cortex (Houdé et al., 2010; see also Kaufmann et al., 2011). The recruitment of the frontal network could be one of the first cortical systems that associate non-symbolic numerosities and symbolic numbers (Nieder and Dehaene, 2009; Opfer and Siegler, 2012). A fundamental region implicated in numerical cognition in adults and children, the intraparietal sulcus (IPS), is increasingly involved during childhood due to academic learning (Rivera et al., 2005; Nieder and Dehaene, 2009; Zamarian et al., 2009). In adults, Aydin et al. (2007) examined the effect of mathematics expertise in two groups and showed less GM in the parietal regions of non-experts. In children, structural MRI studies are scarce and have only investigated individual differences by comparing dyscalculic and control children (Isaacs et al., 2001; Molko et al., 2003; Rotzer et al., 2008; Rykhlevskaia et al., 2009). The results indicate that dyscalculic children present with less GM, especially in parietal and frontal brain areas such as the IPS or the middle and inferior frontal gyri. Inter-individual differences are a rich source of information to link human cognition to cerebral anatomy (Kanai and Rees, 2011). However, we know of no study that has directly examined this relationship in typical development.

The aim of our study is to compare typically achieving schoolchildren of the same age with different levels of expertise in numerical cognition. For this comparison, we used the Voxel-Based Morphometry (VBM) method (i.e., an analysis that allows the detection of volumetric brain variations between two groups of subjects and their localizations after separating gray and white matter from brain anatomical MRI images, see Ashburner and Friston, 2000). We searched for possible neuroanatomical differences between the numerical cognition performances of two groups of 10-year-old schoolchildren, only differing in their numerical transcoding proficiency (i.e., the representation of numerical magnitude, the core of number processing, and its link to numerical symbols). We used two standardized subtests (i.e., number line task and visual estimation of quantities task) from the ZAREKI-R, a mathematical cognition battery (Von Aster and Dellatolas, 2006) that allows assessment of the transcoding proficiency of the children. These two subtests address the representation of numerical magnitude and its link to numerical symbols: the transcoding processing from the symbolic system to the analog system (number line task) and the transcoding processing from the analog system to the symbolic system (visual estimation of quantities task). According to developmental maturation data (Gogtay et al., 2004), one would expect that the higher level group would present a loss of GM in some brain areas due to a more advanced maturation in agreement with a “synaptic pruning” phenomenon (i.e., a fundamental neural plasticity mechanism that may underlie selective behavioral specialization, see Edelman, 1993). Alternatively, from an experience-dependent structural changes perspective, one would expect that better performance would depend on an increase in GM. For the first time, our study seeks to contribute to a better understanding of this important open issue regarding the intertwined relationships between maturation, experience, and learning in typical developing children.

## Materials and Methods

### Participants

Twenty-two children recruited from schools in Caen (Calvados, France, forth grade, *N* = 14, fifth grade, *N* = 8) participated in this study (mean age ± SD, 10 years ± 7 months; age range: 9.2–11.1 years; 11 girls; 19 right handed). They had no history of neurological disease and no cerebral abnormalities as assessed by their T1-weighted MRI. The local ethics committee (CPP Caen Nord Ouest III, France) approved the study. Written consent was obtained from the parents and the children themselves after detailed discussion and explanations.

### Standardized measure of numerical transcoding proficiency

The children passed a whole test battery that was individually administered at school, including the Neuropsychological Test Battery for Number Processing and Calculation in Children (ZAREKI-R, Von Aster and Dellatolas, 2006, French version). We focused on two tasks that allow assessment of numerical transcoding proficiency: number line task and visual estimation of quantities task. The duration of the two paper-pencil tests was approximately 10 min, and the children were first presented with the number line task and then with the estimation of quantities task. These subtests address the representation of numerical magnitude and its link with numerical symbols: transcoding from the analog system to the symbolic system and *vice versa*. In the task requiring estimation of visual quantities, the child had to verbally estimate a number of objects that were quickly presented (dots, glasses, balls). For example, 14 dots were presented on a sheet of paper for 2 s, and the child had to estimate how many dots were present. The child could obtain a maximum score of 5 points (one point was given for each correct response). In the number line task, the child had to place a number (oral or visual presentation) on a vertical line ranging from 0 to 100. For instance, the child had to indicate with a drawing pencil on a sheet of paper where the number “56” was on the vertical line marked from 0 to 100. The child could obtain a maximum score of 24 points (two points were given for each correct response). We then transformed the raw scores for the two tasks into *Z*-scores (see Earnst and Kring, 1999; Foy and Mann, 2013). To detect group differences based on numerical transcoding proficiency, a composite measure was computed by adding the *Z*-scores for these two tasks across all children. To verify that our two groups differed only in numerical transcoding proficiency, we also assessed mental calculation (ZAREKI-R, mental calculation task, Von Aster and Dellatolas, 2006, French version), reading (“L’Alouette-R,” Lefavrais, 2005) and working memory (WISC-IV, digit span subtest, Wechsler, 2005, French version) abilities.

### MRI acquisition

The children were familiarized individually for MRI investigation in a half-hour-long session at school the day before the MRI. This session consisted of a “statue game” in which they needed to stay as still as a statue in a toy tunnel imitating the MRI scanner and its technological environment, including the recorded noises of all MRI sequences, cardboard head coil and medical tape on the forehead (Houdé et al., 2011). The same familiarization process was repeated just prior to the experiment. Anatomical scans were acquired on a 3T MRI scanner (Achieva, Philips Medical System, Netherlands) at the Cyceron biomedical imaging platform (www.cyceron.fr) in Caen (Calvados, France) using a 3D T1-weighted spoiled gradient (FOV: 256 mm; isotropic voxel size: 1.33 mm; 128 slices; matrix size 192 × 192 voxels; 5 min 7 s duration). Data were acquired while the children passively watched a cartoon on an MRI-compatible screen. The sedative effect of audio/visual systems on children in MRI scanners has been demonstrated; it reduces head motion, provides a positive experience, and decreases wait times (Lemaire et al., 2009).

### Data analysis

The T1 scans were spatially normalized and segmented into three tissue classes (i.e., gray matter, white matter, and cerebrospinal fluid) with SPM5 (statistical parametric mapping) software (Wellcome Department of Cognitive Neurology, London, UK, www.fil.ion.ucl.ac.uk/spm) implemented in Matlab version 7.4 (Mathworks, Inc., Natick, MA, USA) using a specific template based on the T1-weighted scans (55 MR scans of typically developing children aged 5–12 years acquired with the same MRI scanner and including children’s images acquired in the present study). A factorial VBM analysis (Ashburner and Friston, 2000) comparing the two groups of children was performed using SPM5 software on normalized, modulated, and 8 mm FWHM Gaussian-smoothed GM volumes. This analysis included a total brain volume correction for each subject, brain volume values being included as a global calculation in the SPM model. The results reported thereafter were obtained for *p* values corresponding to *p* < 0.001 uncorrected with clusters with a minimum of 80 voxels. VBM analysis was performed using two-sample *t*-tests contrasting the two groups including age as covariate.

## Results

### Behavioral results

The children were grouped according to their numerical transcoding proficiency composite *Z*-score. Children were included in the low numerical transcoding (LNT) group if their composite *Z*-scores were negative and in the high numerical transcoding (HNT) group if their composite *Z*-scores were positive, with 10 and 12 children, respectively. According to ZAREKI-*R* standardization (number line task mean ± SD = 17.63 ± 3.73; and visual estimation of quantities task mean = 3.98 ± 1.01), all the children in the LNT group exhibited at least one score in the second quartile (number line task = 15–18.25; and visual estimation of quantities task = 3–4), while all the children in the HNT group displayed at least one score in the third quartile (number line task = 18.25–22; and estimation task = 4–5). Importantly, the mean performances of the LNT group on both tasks were in the second quartile, while the mean performances of the HNT group were in the third quartile. The composite scores differed significantly between the two groups [*t* (20) = −7.58, *p* < 0.0001, *d* = −3.39] but importantly, the groups did not differ according to sex (*p* > 0.19, Fischer’s exact test), handedness (*p* > *1*, Fischer’s exact test), age [*t* (20) = −0.33, *p* > 0.74, *d* = −0.15], or months of education [*t*(20) = 0.30, *p* > 0.76, *d* = 0.13]. Moreover, they did not differ according to mental calculation [*t*(20) = 0.27, *p* > 0.79, *d* = 0.12], reading [*t*(20) = −0.86, *p* > 0.40, *d* = −0.39], and working memory [*t*(20) = −0.67, *p* > 0.50, *d* = −0.30] performances (see Table 1). Note that there were equal numbers of fifth grade children in the LNT group (*n* = 4) and in the HNT group (*n* = 4).

**Table 1:** **Participant characteristics by group**.

	LNT (*N* = 10)		HNT (*N* = 12)		*p* Value
	Mean	SD	Mean	SD	
Age (months)	120	7.5	121	7.0	ns
Number line score	16.9	3.1	20.3	2.0	0.005
Visual estimation score	3.3	1.2	5.0	0	0.0005
Composite *Z*-score	−1.4	0.9	1.2	0.6	0.0001
Calculation score	37.8	4.1	37.3	5.3	ns
Reading scores	267.6	93.8	298.4	73.1	ns
Working memory scores	15.9	2.6	16.2	4.6	ns

*LNT, low numerical transcoding; HNT, high numerical transcoding*.

*SD, Standard deviation, ns, *p* > 0.05*.

### VBM results

**Figure 1:**
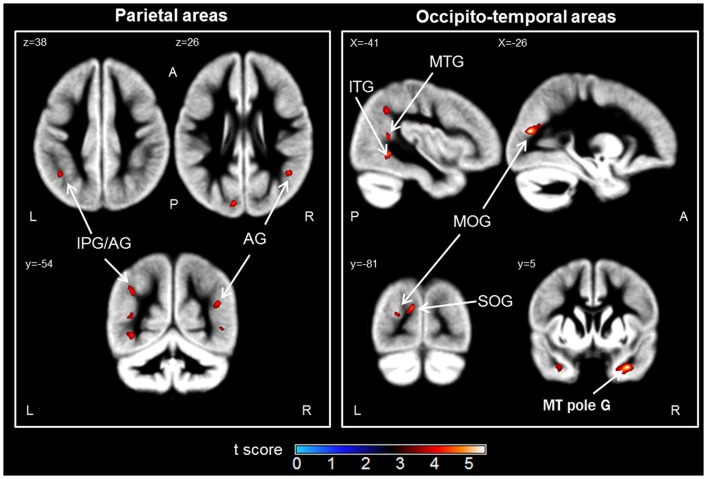
**Brain regions in which the low numerical transcoding (LNT) group showed significantly less gray matter volumes compared to the high numerical transcoding (HNT) group (two-sample *t*-test results for HNT > LNT contrast)**. Notes: significant differences (*p* < 0.001 uncorrected) are displayed on a mean Gray Matter (GM) template (average of 55 normalized GM scans). AG, angular gyrus; IPG, inferior parietal gyrus; MOG, middle occipital gyrus; SOG, superior occipital gyrus; MTG, middle temporal gyrus; MT pole G, middle temporal pole gyrus; ITG, inferior temporal gyrus; L, Left; R, Right; A, Anterior; P, Posterior.

The contrast analysis demonstrated that the children in the LNT group had less GM volume than the children in the HNT group in a network including parietal, temporal, and occipital cortices (see Figure 1 and Table 2). The identified parietal regions were the right angular gyrus and left inferior parietal gyrus along the intraparietal sulcus extending to the angular gyrus. The temporal regions were the right pole of the middle temporal gyrus extending to the parahippocampal and fusiform gyri, and the left superior, middle and inferior temporal gyri. Finally, the occipital regions included the left middle occipital gyrus and the cuneus extending to the superior occipital gyrus. The children in the LNT group did not have significantly larger GM volumes than the children in the HNT group in any brain region.

**Table 2:** **Anatomic localization (AAL, Anatomical Automatic Labeling), localization extent and Montreal Neurological Institute (MNI) coordinates of brain areas that showed less gray matter volumes in children**.

Anatomical localization (AAL)	Hemisphere	Number of voxels	MNI Coordinates			*z-Value*
			*X*	*Y*	*Z*	
**HNT > LNT**
Angular gyrus	R	122	45	−53	26	3.56
Inferior parietal gyrus (intraparietal sulcus), angular gyri	L	118	−41	−54	38	3.43
Middle temporal pole, parahippocampal, fusiform gyri	R	855	34	5	−35	4.17
Middle and inferior temporal gyrus	L	140	−40	−52	−4	3.73
Superior temporal gyrus	L	89	−66	−10	2	3.64
Middle temporal gyrus	L	86	−41	−52	12	3.51
Middle occipital gyrus	L	342	−26	−77	18	4.17
Cuneus and superior occipital gyrus	L	242	−10	−83	23	3.94
**HNT < LNT**
No significant difference

*L, left; R, right*.

## Discussion

The relationships between children’s numerical transcoding proficiency and cerebral anatomy in the typical developing brain were, to our knowledge, investigated for the first time here. To address this open issue, we used the standard VBM approach to detect differences in GM in two groups of 10-year-old, typically achieving schoolchildren varying in level of transcoding proficiency.

Our results demonstrated that children with low numerical transcoding have less GM volume in parietal and occipito-temporal areas than children with high numerical transcoding. Parietal regions are crucial in number cognition (Dehaene et al., 2003; Ansari, 2008; Nieder and Dehaene, 2009). According to the triple code model of number processing (Dehaene, 1992; Dehaene and Cohen, 1995; Dehaene et al., 2003), numbers are represented in three distinct systems that serve different functions and that have distinct functional neuroarchitectures related to performance on specific tasks: an analog, a verbal, and a visual system. In this model, parietal areas would be the core of the analog system, corresponding to the representation of the quantity. At the neuroanatomical level, structural differences related to competences have been identified in parietal areas in atypical development such as dyscalculia (Isaacs et al., 2001; Molko et al., 2003; Rotzer et al., 2008; Rykhlevskaia et al., 2009). Indeed, dyscalculic children, known to present deficits in the “number sense” (i.e., difficulties in understanding a quantity and in number sense access), exhibited GM loss in bilateral parietal areas compared to control subjects. Moreover, one study examined the effect of mathematics expertise between two adult groups (Aydin et al., 2007). The data indicated a GM decrease in parietal regions in non-expert. Our results are in agreement with these studies. A poorer performance seems be related to lower GM volume in parietal areas even in typically achieving children.

It is known that three circuits co-exist in the parietal region (Dehaene et al., 2003): (i) the bilateral horizontal segment of the intraparietal sulcus (IPS) supports quantity processing, (ii) the left angular gyrus (AG) supports manipulation of numbers in verbal form, and (iii) the bilateral posterior superior parietal lobe supports spatial attention processes. We found GM volume changes in the left IPS, a region that has previously been identified in our number processing functional meta-analysis (Houdé et al., 2010). This is a core parietal region implicated in numerical cognition (i.e., number sense) and in magnitude judgment. We also observed GM changes in bilateral AG, which is functionally involved in the transition from quantity-based processing to automatic fact retrieval (Dehaene et al., 1999; Grabner et al., 2007, 2009a; Ischebeck et al., 2007), the processing of digits (Price and Ansari, 2011), or the mapping between a symbol and its referents (Ansari, 2008; Grabner et al., 2009b). Interestingly, the AG has also been involved in non-symbolic number processing (Göbel et al., 2001; Kaufmann et al., 2011). Furthermore, competence-related differences were observed in functional activations of the left AG depending on the level of mathematic competence (Grabner et al., 2007, 2009b). The higher-competence group showed a stronger activation during an arithmetic task. We hypothesize that these functional modulations would be based on structural differences. In our opinion, the GM volume variations observed in the left IPS and the bilateral AG suggest specific expertise-dependent plasticity of these regions. Our tasks assessed transcoding between the analog system and symbolic system, such as Arabic digits or number words. For instance, when the experimenter tells him/her a number, the child must place it on a number line. Children who are less successful in these tasks are less efficient in the transcoding between analog and symbolic systems. Lower performance in the representation of the quantity and in the transcoding of one code to another is associated with less GM volume the IPS and AG brain areas, which are known for their roles in the analog system and in the symbolic manipulation of numbers.

Our study also revealed a GM change according to the level of transcoding proficiency in the left middle and superior temporal gyri, which are involved in the number verbal system previously described in the triple code model of number processing. We also observed GM modulation in such regions of the ventral visual stream as the cuneus, the superior occipital gyrus, the occipital lateral gyrus and the inferior temporal gyrus. While most studies of numerical cognition have focused on the dorsal stream, little attention has been paid to the ventral visual stream, which is thought to support recognition and discrimination of visual objects, such as digits (Milner and Goodale, 2008; Rosenberg-Lee et al., 2011). The temporal lobe seems to play a role in the link between symbols and the meaning of numbers (Jefferies et al., 2005; Julien et al., 2010). The right anterior temporal cortex, known for its involvement in semantic processing (Visser et al., 2010), also showed GM volume modulation according to the level of transcoding proficiency. Interestingly, Rykhlevskaia et al. (2009) found abnormalities in this area in dyscalculic children compared to control subjects and suggested that it may be an additional fundamental locus in the numerical cognition, perhaps as the semantic memory representation.

Finally, we did not observe GM differences in frontal areas. As suggested by Nieder and Dehaene (2009) and Opfer and Siegler (2012), the recruitment of the frontal network could be one of the first cortical areas that associates non-symbolic numerosities and symbolic numbers. With age, there may be a shift from the frontal cortex to the parietal cortex to perform numerical tasks (Rivera et al., 2005; Houdé et al., 2010). Here, the numerical tasks were relatively easy for 10-year-old children. They learn the association between a quantity and its symbol (number words then digits) from the start of their schooling. The link is already formed, even if it is imperfect in some children. Thus, for this type of task at this age, GM differences observed in the posterior regions could better explain the present results than differences in anterior areas. Indeed, as suggested by Butterworth et al. (2011), a link between the occipito-temporal and the parietal cortex is required for transcoding number symbols onto the numerosity representation.

Taken together, our data may reflect the structural modulation relating to numerical transcoding proficiency in the brain areas dedicated to the transcoding between a quantity and its symbol. One could wonder why the GM volume increases rather than decreases with performance. Cerebral maturation is characterized by a loss of GM with age due to a neural plasticity mechanism called “synaptic pruning” (Gogtay et al., 2004). However, as suggested by Kanai and Rees (2011), GM microstructure could be different, such as GM with more neurons (expertise) or GM with fewer synapses (maturation). Our results with typically developing children highlight this paradoxical period during which the effects of maturation and academic learning are strongly intertwined. Thus, we suggest that intensive learning during schooling could modulate children’s brain structure and lead to increased GM volume with performance, as has been observed in adults (Draganski et al., 2004, 2006; Aydin et al., 2007). However, further studies will be necessary to disentangle the intertwined relationships between maturation and learning in the developing brain.

In conclusion, our study, linking numerical transcoding proficiency with brain structure in schoolchildren, contributes to a better understanding of how mathematics learning can modulate brain regions involved in the numerical transcoding. Even if our results were consistent with the numerical cognition literature, it is important to consider them with caution because of the limited number of participants and the use of non-corrected thresholds. It will be interesting to examine the impact of pedagogical interventions on structural brain variations using a neuroeducational approach. For instance, future work will investigate the potential brain variations induced by the use of numerical games, which appear to have a positive effect on number sense access in populations at risk for mathematical difficulties (Wilson et al., 2009). Neuroeducation could undoubtedly offer an exciting new method for future studies to form a link between education and developmental cognitive neuroscience in regard to mathematics learning.

## Conflict of Interest Statement

The authors declare that the research was conducted in the absence of any commercial or financial relationships that could be construed as a potential conflict of interest.
